# Extraction of nano-crystalline cellulose for development of aerogel: Structural, morphological and antibacterial analysis

**DOI:** 10.1016/j.heliyon.2023.e23846

**Published:** 2023-12-18

**Authors:** Yash Vishnoi, Alok Kumar Trivedi, M.K. Gupta, Harinder Singh, Sanjay Mavinkere Rangappa, Suchart Siengchin

**Affiliations:** a Department of Mechanical Engineering, Motilal Nehru National Institute of Technology Allahabad, Prayagraj 211004, U.P. India; b Department of Chemical Engineering, Motilal Nehru National Institute of Technology Allahabad, Prayagraj 211004, U.P. India; c Natural Composites Research Group Lab, Department of Materials and Production Engineering, The Sirindhorn International Thai-German Graduate School of Engineering (TGGS), King Mongkut's University of Technology North Bangkok (KMUTNB), Bangkok, Thailand

**Keywords:** Nano-crystalline cellulose, Biomass, Facile approach, Structural properties, Antibacterial properties, Aerogel

## Abstract

In the present decades, nanocellulose has been very popular in the field of nanotechnology and is receiving much attention from researchers because of its advantageous physicochemical properties, high aspect ratio, and high specific strength and modulus. The available non-eco-friendly conventional methods for the extraction of nano-crystalline cellulose (NCC) use highly concentrated chemicals and are time-consuming as well. The present adopted cost-effective method for the extraction of nano-crystalline cellulose involves minimum usage of chemicals and is environmentally friendly and relatively fast compared to other conventional methods. The nano-crystalline cellulose from sisal (NCC–S) fibers were extracted by steam explosion-assisted mild concentrated chemical treatments followed by mechanical grinding. The Dynamic light scattering (DLS) and Transmission electron microscopy (TEM) characterization confirmed the size of extracted NCC-S. A high aspect ratio was observed as 19.23, which signifies it could be a promising reinforcing material in developing nanocomposites for advanced applications. An increase in crystallinity and the removal of amorphous materials for NCC-S were confirmed by X-ray diffraction (XRD) and Fourier-transform infrared spectroscopy (FTIR) analysis, respectively. Antibacterial study shows that NCC-S did not show any antibacterial properties against *E. coli* and *S. aureus*. The calculated yield of extracted nanocellulose was about 50 %. The aerogel with a porosity of 95.1 % and a density of 0.075 g/cm^3^ was prepared by vacuum freeze-drying method using extracted nanocellulose and chitosan. The cross-linking network structure and thermal stability of the aerogel were also confirmed by FTIR and TGA analysis respectively.

## 1 Introduction

Nanocellulose is a form of cellulose in the nano range which can be derived from different plant fibers [[1], [2], [3], [4], [5], [6]]. Nanocellulose is mainly categorized into three different categories such as cellulose nanofibrils (CNF), cellulose nanocrystals (CNC), and bacterial nanocellulose (BNC). The CNF can be 20–50 nm wide and 500–2000 nm long [7,8]. CNC has a more elongated rod-like structure and less flexibility than CNF due to its increased crystallinity [9,10]. CNCs have a wide variety of geometrical dimensions with diameters ranging from 5 to 50 nm and lengths ranging from 100 to 500 nm [[11], [12], [13]]. Nanocellulose is a bio-degradable, non-toxic, eco-friendly and renewable resource with good properties like high surface area, low density, high thermal stability, good mechanical and optical properties, and bio-compatibility [14,15]. It has a high aspect ratio and its specific young's modulus is 3.4 times higher than steel [5]. It has been utilized for a wide range of applications such as nanocomposite materials, biomedical products, wood adhesives, supercapacitors, batteries, electroactive polymers, continuous fibers and textiles, separation membranes, antimicrobial films, paper products, and many more [7,[16], [17], [18]].

Nanocellulose shows a very good antibacterial potential against various kinds of bacteria as reported in many previous studies [[2], [3], [4], [5], [6]]. Gond et al. [3] extracted nanocellulose from sugarcane bagasse and investigated their antibacterial properties against *Bacillus* and *E. coli* bacteria by disc diffusion method. It was observed that extracted nanocellulose shows good antibacterial properties against both these bacteria. In another study, a higher inhibition zone was observed against *E. coli* than the *Bacillus* which signifies that nanocellulose is more sensitive to *E. coli.* Errokh et al. [6] produced hybrid nanocellulose coated with silver particles and investigated their antibacterial properties against *E. faecalis* and *Micrococcus (Gram*^*+*^*)* and *E. coli (Gram*^–^*)*. This coated nanocellulose exhibited enhanced antibacterial properties against these bacteria. They proposed these coated nanocellulose particles as reinforcement materials for biocomposites film that can be used for various applications, including packaging, coating, etc. Similarly, Faghihi et al. [19] also studied the antibacterial property of poly (ether-amide)/silver nanocomposites (PANCs) and observed good antibacterial properties against *E. coli*.

Sisal fibers contain cellulose (50–74 %), lignin (8–11 %), hemicellulose (10–14 %), pectin (1 %) and wax (2 %) [20]. The main reason behind choosing sisal fibers is their high cellulose content which can help to extract a higher quantity of nanocellulose. The sisal plant has an average life span of around 7–10 years and typically produces 220–270 commercially useable leaves. There are about 900–1100 fibers in a single leaf. The plant can be found in different parts of the world like India, China, Nepal, Africa, Burma, Mexico, etc. Looking inside the cross-section of sisal fibers, we found 100–200 bundles of single cells joined by natural gums. Sisal fibers do not have a uniform dimension or perfectly circular cross-section. Longitudinally, the fibers are straight and without crimp looks like a cylinder [21].

Nanocellulose from sisal fibers was extracted using various conventional chemical treatments followed by mechanical treatment. Moran et al. [22] extracted cellulose from sisal fibers using two different chemical methods and obtained average size' 22.0 ± 9.2 μm and 9.1 ± 2.1 μm by first and second methods respectively. The crystallinity of the microcellulose obtained from both methods was approximately 75 %. Trifol et al. [23] extracted CNF from sisal fibers using strong alkali treatment (two times with 2 & 10 w/v% NaOH), bleaching (25 wt % NaClO_2_) and followed by acetylation (1:18, g/mL). The extracted CNF's average dimensions were 27 ± 13 nm in diameter and 658 ± 290 nm in length. The crystallinity of the CNF was observed as 84.2 %. This method utilized high concentrations of chemicals and was a bit time-consuming. Therefore, such type of process could not be considered an efficient and eco-friendly method for the extraction of nanocellulose. Similarly, Jain et al. [24] extracted CNC from the sisal fibers by highly concentrated chemical treatments including alkali treatment (10 wt/v % NaOH for 2 h), bleaching (10 wt % sol. for 18 h) and acid hydrolysis (40 wt % H_2_SO_4_ for 4 days) and followed by centrifugal process.

After a detailed literature survey, it can be observed that the reported conventional methods for the extraction of nanocellulose consume huge amounts of chemicals that are very hazardous to the environment and society [[14], [15], [16], [17], [18], [19], [20]]. Steam explosion is a pre-treatment process to extract cellulose from plant biomass. It improves the extraction rate as compared to other pre-treatment processes. It can be either done alone or in combination with other processes. The main benefits of using this method are low energy consumption, low chemical requirements, low environmental effects, and low-cost. After a comprehensive literature survey, it was observed that steam explosion could be an efficient approach for the extraction of nanocellulose with minimum consumption of chemicals in an optimum time [[25], [26], [27], [28], [29]].

This pre-treatment is basically based on heating the materials for a short term at high pressure (0.69–4.83 MPa) and temperature (160–260 °C), and then the pressure is abruptly reduced, which makes the materials feel an explosive decompression [30,31]. Different types of lignocellulosic biomass had undergone this pre-treatment as already reported: cotton [32], wheat straw [33], bamboo [34], and pineapple leaf fibers [25]. Jeoh et al. [32] analyzed the effects of steam explosion on cotton gin waste for ethanol production. It was reported that steam-exploded fibers improved enzyme hydrolysis treatment. The ethanol yield percentage was highest for steam-exploded biomass compared to untreated fibers because of the high degradation and solubilization of the fibers. Kaushik et al. [33] also extracted cellulose nanofibrils from wheat straw biomass using steam explosion-assisted chemical treatment. Shao et al. [34] investigated the effects of steam explosion on the chemical properties of the bamboo internode biomass. The steam explosion process cleaved the major hemicellulose content in the bamboo. They reported that steam explosion causes defibrillation on the bamboo internode leads to significant changes in their chemical structure. Similarly, Cherian et al. [25] reported that steam explosion-assisted chemical treatment was an effective method for defibrillation and depolymerizing the pineapple leaf fibers. Tanpichai et al. [26] employed steam explosion method for the extraction of micro-cellulose from pineapple leaves. This study suggested that steam explosion-assisted chemical treatment helped in the removal of hemicellulose and lignin from the fibers and facilitated the extraction process with enhanced cellulose content. It was also noticed that with an increase in the steam pressure and treatment cycle, the fibers get more defibrillated hence decreased the diameter and length of the cellulose was obtained.

Aerogels are highly porous and ultralight materials derived from the gel by replacing the liquid in the suspension with the air. The first aerogel was successfully prepared by Samuel Kristlerin around 1931 from wet silica gel through a supercritical drying method [27]. The general properties of the aerogels are high porosity, low density, high specific surface area, and low thermal conductivity. Nanocellulose-based aerogels are superior to polymer and inorganic aerogels as they are biocompatible, biodegradable, non-toxic, produce minimum carbon footprint, and are economical [28]. Aerogels are very popular among researchers and have been proposed for several advanced applications such as thermal insulation, energy storage, acoustic and separable membrane, and filters. Nargatti et al. [29] recently published a review study on the application of aerogel as an electrode for supercapacitors. This study emphasizes the development of supercapacitors from renewable and sustainable resources. The mechanical strength of the aerogel made from nanocellulose is insufficient to make a robust electrode for energy storage devices. The porous structure of the electrode increases the ion diffusion rate in an electrolyte which provides high current density, which is essential for energy storage devices [35].

The aerogel prepared from nanocellulose only does not exhibit good adsorption properties. But, its adsorption behavior can be enhanced significantly by preparing cellulose based composite aerogel. The porous structure of aerogel has a vital role in the adsorption properties of aerogel. The aerogel with mesoporous structure has a good adsorption effect. Shaheed et al. [36] fabricated the aerogel from nanocellulose (CNC & CNF) and its derived composites (Cu-BTC/CNC & Cu-BTC/CNF) and investigated their adsorption properties. It was observed that Cu-BTC/CNF composite aerogel shows excellent adsorption behavior for organic dye (Congo Red). In contrast, pure CNF aerogel didn't show absorption behavior for the same organic dye. This study also reveals that pure CNF aerogel and Cu-BTC/CNF composite aerogel act as monolith standing solid reducers that can convert the permanganate ions of water to magnetic dioxide without releasing any by-products. Similarly, Zhou et al. [37] fabricated the aerogel from pure nanocellulose and solvent-free alumina particles loaded with nanocellulose and studied their adsorption capacities against oil and organic solvents. It was observed that alumina particles loaded nanocellulose shows the highest adsorption properties (64.83 ± 2.25 to 117.65 ± 5.68 g/g) as compared to aerogel fabricated from pure nanocellulose (48.49 ± 1.01 to 87.03 ± 0.046 g/g).

Gu et al. [38] fabricated nanocomposite aerogel by mixing nano chitosan and reduced graphene oxide to improve the oil absorption capacity of the aerogel. They observed that the aerogel fabricated with the mixing of 0.1 wt % nano chitosan and 0.05 wt % nanocellulose had a minimum density of 9.3 mg/cm^3^ and high absorption capacity. Similarly, Rizal et al. [39] also fabricated scaffolds from nanocellulose/chitosan aerogel through high-pressure homogenization and freeze-drying method. From this investigation, it was observed that aerogel prepared from pure cellulose nanofibrils (CNF) has high porosity and low density, but at the same, it was not sufficiently mechanically robust. Based on this characterization, this aerogel can be used as a scaffold for tissue engineering in biomedical applications. Chitosan is a natural polysaccharide with the chemical formula poly-β-(1,4)-2-amino-2deoxy-glucopyranose. It is prepared by the chitin polymer's deacetylation and enzymatic degradation process [40]. Chitosan-based composite aerogels are new sustainable natural biopolymers for the development of sustainable biocomposites due to their excellent biocompatibility, biodegradability, nontoxicity, antibacterial behavior, abundance, and low cost [39,41]. Gaspari et al. [42] enhanced the water resistance properties of plam fibers by applying a coating blend of chitosan-AESO (acrylate epoxidized soybean oil). Tanpichai et al. [43] also carried out a review study on nanocellulose in nanocomposite foams and multifunctional applications. They highlighted that uniform dispersion of nanocellulose in the less hydrophilic polymer is very challenging. This issue could be overcome by mixing the nanocellulose with other nanoparticles such as nano graphene particles, carbon nanotubes (CNT) and zeolite to improve the performance of porous nanocomposite materials.

In the present work, CNC was extracted from sisal fibers by an efficient approach of steam explosion assisted with low-concentration acid followed by mechanical grinding subjected to its characterization in terms of morphological, structural, crystalline and antibacterial analysis. The objective behind adopting the present method was to minimize the consumption of chemicals to reduce harmful impacts on the environment and reduce extraction time. The extracted CNC was further utilized to prepare nanocellulose/chitosan aerogel. The surface morphology, porosity, density and thermal properties of the prepared aerogel were also investigated.

## 2 Materials & methodology

### 2.1 Materials

Untreated sisal fibers used in this work were purchased from the Women's development organization, Dehradun, Uttrakhand, India. Other reagents, sulphuric acid, sodium hydroxide, sodium hypochlorite, and chitosan, were purchased from Uma Scientific Traders, Prayagraj, Uttar Pradesh, India. This work used sodium hydroxide and sulphuric acid was 98 % pure, and sodium hypochlorite had 10–12 % chlorine. Chitosan has a molecular weight of approximately 20 kDa and a degree of deacetylation ≥80 %. Deionized water used in the process was collected from the Centre for Interdisciplinary Research lab, MNNIT Allahabad, Prayagraj, Uttar Pradesh, India.

### 2.2 Methodology

#### 2.2.1 Extraction of nano-crystalline cellulose

**Fig. 1:**
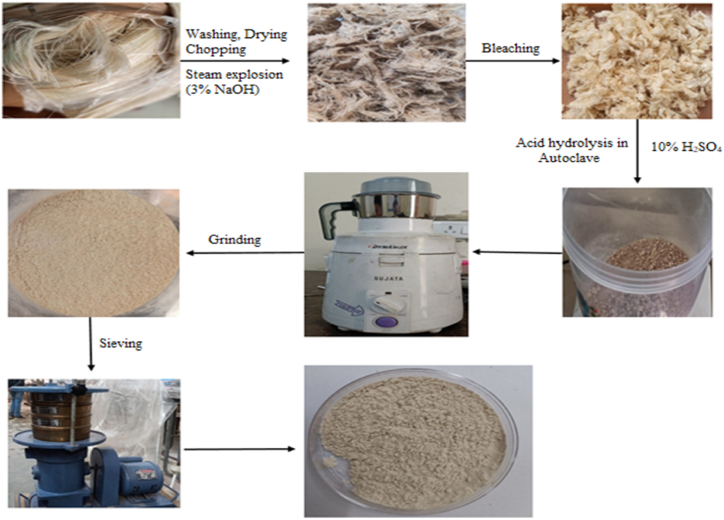
Extraction process of nano-crystalline cellulose using steam explosion assisted with chemo-mechanical treatmentfrom sisal fibers.

The methodology used for extracting nanocellulose is shown in Fig. 1. Firstly, the untreated sisal fibers were washed with deionized water to remove dirt and dried in a hot oven at 60 °C for 12 h. Consequently, the untreated sisal fibers were chopped into 2 inches of small fibers. After that, chopped fibers were treated with 3 % sodium hydroxide solution in an autoclave at 15psi (gauge) for 1 h and then steam was removed suddenly to perform a steam explosion. This step was repeated twice. After this, the treated fibers were again washed with deionized water until pH 7 was not maintained. The treated fibers were bleached with sodium hypochlorite solution at 60 °C with 1:3 fibers to bleaching solution ratio for 4 h in a hot air oven. Again, the fibers were washed with deionized water to maintain pH 7. Acid hydrolysis of fibers was performed with 10 % sulphuric acid solution in an autoclave at 15 psi in the ratio of 1:10 of fibers and acid for 1 h. Fibers were again washed with deionized water until pH 7. Fibers were dried in a hot air oven for 24 h at 60 °C. Now, the grinding process was done at 1500 rpm and particles were sieved several times using a sieve shaker to get a uniform size of nanocellulose. Some notations have been used for different types of fibers: USF = untreated sisal fibers, SFT1 = sisal fibers after the steam explosion in alkaline medium, SFT2 = sisal fibers after bleaching treatment, and NCC–S = extractednano-crystalline cellulose.

#### 2.2.2 Preparation of aerogel

**Fig. 2:**
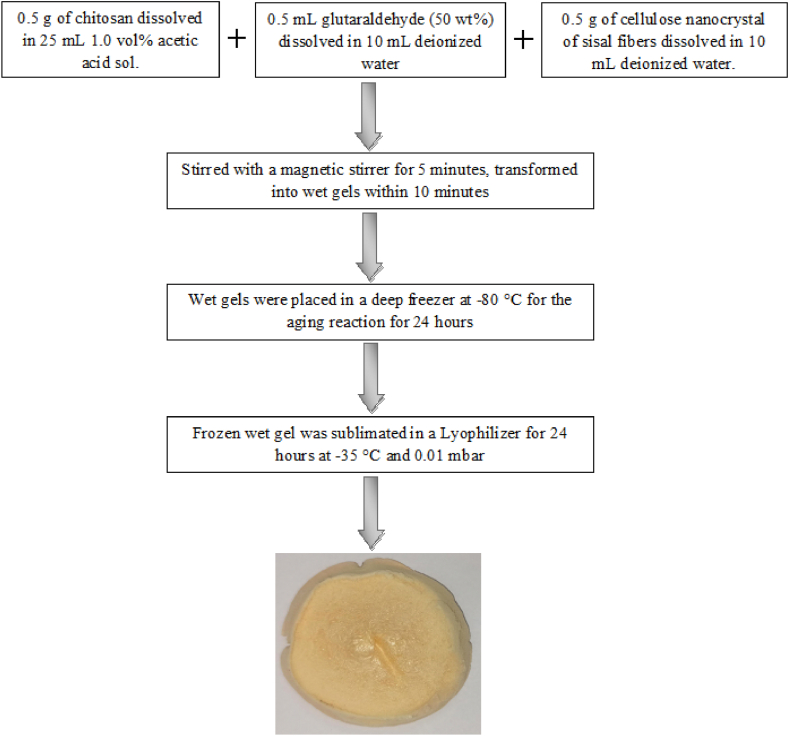
Preparation of aerogelusing freeze-drying method from NCC-S and chitosan.

The methodology utilized to prepare aerogel from NCC–S and chitosan is shown in Fig. 2. In this process, The chitosan (0.5 g) was dissolved in 25 mL of 1.0 vol% acetic acid solution, 0.5 mL glutaraldehyde (50 wt%) dissolved in 10 mL deionized water and NCC–S (0.5 g) dissolved in 10 mL deionized water. These three solutions were mixed and stirred vigorously with a magnetic stirrer for 5 min, then progressively transferred into wet gels within 10 min. Wet gels were placed in a deep freezer at −80 °C for the aging reaction for 24 h. The frozen wet gels were sublimated for 24 h at temperature and pressure of −35 °C and 0.01 mbar, respectively. The Lyophilizer is a sophisticated instrument that operates at desired temperature and pressure. Its function is to convert the frozen solution's ice to vapor directly, known as sublimation. The porous space of gel is occupied with air, subsequently, this porous structured material is known as aerogel. After the sublimation wet gels were successfully converted into aerogels, as shown in Fig. 2.

## 3 Characterization of nanocellulose

### 3.1 Yield measurement

The yield of extracted nanocellulose was calculated by the following gravimetric method. The percentage yield was calculated by the following Equation (1) [34].

(1) $$\text{Yield}\;\left(\%\right)=\frac{w_{2}}{w_{1}}\times 100$$

where $w_{2}$ = final weight of extracted dry nanocellulose and $w_{1}$ = weight of initial dry untreated sisal fibers.

### 3.2 Dynamic light scattering (DLS)

DLS has been used to determine the particle size at the micro or nano level, size distribution, diffusion coefficient and hydrodynamic radius. During this analysis, particles of nanocellulose were dispersed adequately in deionized water with the help of ultrasonication. Their size was calculated by the dynamic light scattering (DLS) (model: Microtrac, Nanotrac wave at CIR Lab MNNIT Prayagraj, India).

### 3.3 Transmission electron microscopy (TEM)

TEM was carried out at IISER Mohali (Model: JEM-F200), Punjab, India, to find the size and shape of nanocellulose particles. Further, it was also used to calculate the L/D (aspect ratio) ratio of the nanocellulose particles with the support of Image J software.

### 3.4 Field emission scanning electron microscopy (FESEM)

FESEM was performed at CMTI Bangalore (model: Neon–40), Karnataka, India, to analyze the surface morphology of the fibers after each treatment, including nanocellulose. The samples were made conductive by applying a very thin layer of gold on the surface of the samples.

### **3.5** X–ray diffraction (XRD)

XRD patterns of untreated and treated sisal fibers and NCC–S were recorded using an X–ray diffractometer at IIT Kanpur (Model: Smart lab 3 KW), Uttar Pradesh, India. The diffraction intensities were recorded between 4° and 70° of 2θ at a wavelength of 1.541 × 10^−10^ m. The crystallinity index (Equation (2)) and degree of crystallinity (Equation (3)) were calculated according to Segal's method [44].

(2) $$\text{Crystallinity}\;\text{index}\;\left(\%\right)=\frac{I_{200}‐I_{\text{am}}}{I_{200}}\times 100$$

(3) $$\text{Degree}\;\text{of}\;\text{crystallinity}=\frac{I_{200}}{I_{200}+I_{am}}$$

Where $I_{200}$ = intensity at the crystallographic plane (200), and $I_{am}$ =minimum intensity, which shows an amorphous region.

### 3.6 Fourier transform infrared spectroscopy (FTIR)

It was performed at CMTI (model: Cary 660), Bangalore, Karnataka, India, to analyze the chemical structural changes in nanocellulose and sisal fibers after each stage of treatments. FTIR spectrums were recorded over the 400–4000 cm^−1^ wavelength range.

### 3.7 Antibacterial analysis

The antibacterial analysis of NCC–S was performed against *Escherichia coli* (*E.coli*) and *Staphylococcus aureus* (*S.aureus*) bacteria by the disc diffusion method. The zone inhibition method is used to determine antibacterial activity.

### 3.8 Characterization of aerogel

#### 3.8.1 FESEM

The surface morphology of the aerogel was investigated by FESEM (model: Jeol Japan JSM 7800 F prime) at different magnifications at Indian Institute of Technology, Delhi, India.

#### 3.8.2 Density and porosity

The aerogel's density was determined by measuring the mass and volume of the sample. The porosity of aerogel was estimated by using the following equation (4) []:

(4) $$P=1-\frac{\rho }{\rho_{s}}$$

where ρ indicates the apparent density of aerogel and $\rho_{s}$ indicates the skeleton density of aerogel. $\rho_{s}$ can be calculated by taking the weight ratio to each constituent's density (Equation (5)).

(5) $$\rho_{s}=\frac{1}{\frac{W_{CNC}}{\rho_{CNC}}+\frac{W_{Chitosan}}{\rho_{chitosan}}}$$

where, *W* and ρ are the weight fraction and density of individual constituents of aerogel.

#### 3.8.3 Thermogravimetric analysis (TGA)

Thermal behavior of aerogel was carried out through TGA analysis. The TGA analysis was performed using TGA equipment model: STA–6000, Perkin Elmer at Chemical Engineering Department of Motilal Nehru Notational Institute of Technology Allahabad, Prayagraj. The small amount of samples approximately 4–5 mg was taken in crucible and heated from room temperature to 600 °C with constant heating rate of 10 °C/min in a controlled nitrogen atmosphere. This analysis reveals the change in weight percentage of aerogel with respect to temperature.

## 4 Results and discussion

### 4.1 Yield analysis

To find out the yield percentage, the initial weight of dry sisal fibers was taken 125 g, whereas the final weight of extracted dry NCC–S powder was found to be 61 g. So, the yield percentage of extracted nanocellulose was 48.8 %. Deepa et al. [45] reported a 38.8 % yield for sisal fiber by steam explosion credited to a large time span of acid hydrolysis. In another study, 87 % of the yield for eucalyptus hardwood was reported by irradiation oxidation and organosolvsolubilisationprocess [46]. Steam explosion with NaOH is sufficient to remove hemicelluloses, whereas bleaching removes lignin effectively. Finally, complete lignin, hemicellulose and amorphous region present in the cellulose were removed by acid hydrolysis. The complete removal of noncellulosic components by the present extraction process might be responsible for the present yield percentage.

### 4.2 DLS analysis

**Fig. 3:**
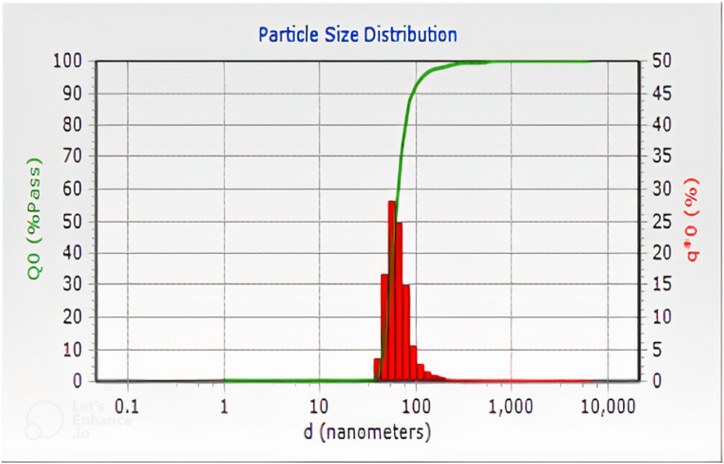
Particle size analysis of nano-crystalline cellulose of sisal fibersfrom DLS analysis.

The DLS method has been widely used to calculate the statistical distribution of particles having the size in the nano-range [3]. It gives information about the change in pass percentage (Q_0_) with respect to the change in particle diameter (d). The particle size distribution of NCC–S is shown in Fig. 3. All the particles having a size in the nano range are credited to a homogenous distribution. This shows the efficiency of the present process used to extract nanocellulose from sisal fibers. The nanocellulose particles of approximately 60 nm have a maximum pass percentage of 55 %, whereas a huge part of the particle group has a size of less than 100 nm. Hence, it can be concluded that particles having a size greater than 100 nm are ignorable. The calculated average size of NCC-S was found to be 61.6 nm. Many researchers have used this method to measure the nano-size of the particles: Gond and Gupta [47] reported a 75 nm diameter of nanocellulose extracted from sugarcane bagasse fibers, Jordan et al. [48] reported an average particle size of 78 nm size of nanofibers extracted from cotton gin waste fibers, Wulandari et al. [49] reported an average diameter of 111 nm for sugarcane bagasse fibers, and Varshney et al. [50] reported 75 nm diameter of nanocellulose extracted from rice husk.

### 4.3 TEM analysis

**Fig. 4:**
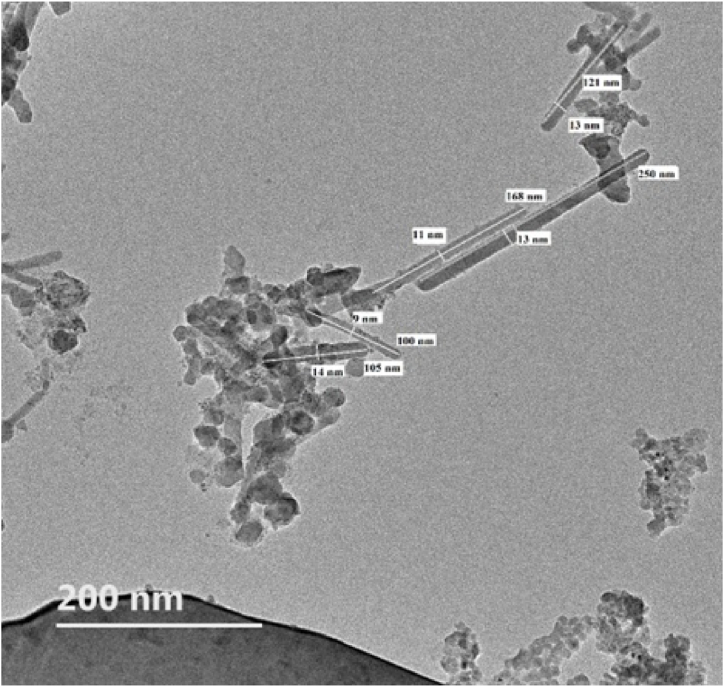
TEM image of nano-crystalline cellulose of sisal fibers through JEM-F200 instrument.

In Fig. 4, the TEM image of the extracted NCC-S is shown. TEM characterization was done to find out the diameter, length, and aspect ratio of the extracted NCC-S with the help of Image J software. From the TEM image, it can be seen that the particles have a long cylindrical rod-like structure. The length of the particles was in the range of 100–250 nm and the diameter observed was in the range of 9–14 nm. With the help of these two data, an aspect ratio for each particle was calculated. After calculating the aspect ratio, the maximum value was found to be 19.23, the minimum was 7.5, and the average was 12.48. The average diameter and length of NCC-S particles were 12 nm and 148.8 nm, respectively. Based on TEM analysis of nanocellulose, similar studies were already reported as aspect ratio from 8.3 to 17 for nanocellulose of rice husk [50], diameter in the range of 11–24 nm, and length in the range of 176 nm–245 nm for CNC [51], and aspect ratio from 10.9 to 12.7 for CNC of kenaf fibers [52].

### 4.4 FESEManalysis

**Fig. 5:**
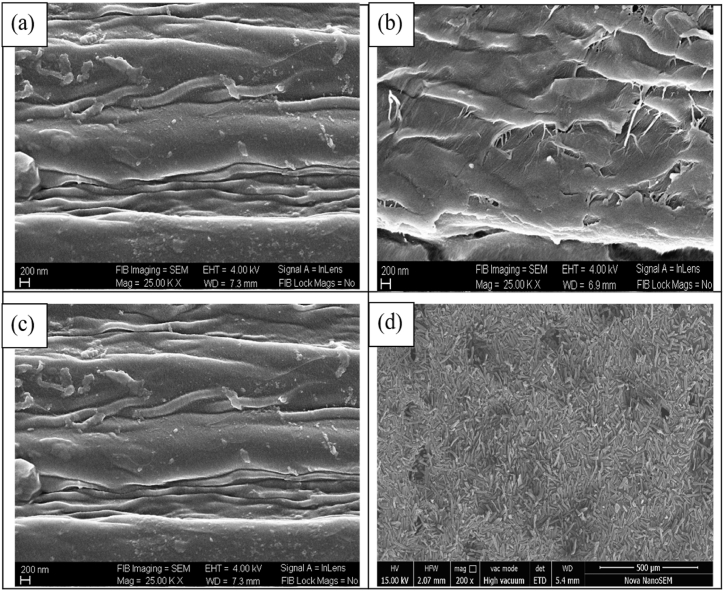
**(a**–**d)**. FESEM images of sisal fibers through Neon-40 instrument at 4 kW EHT: (a) untreated, (b) alkali treated, (c) bleached and (d) nano-crystalline cellulose.

The surface characteristics of untreated and treated sisal fibers and NCC–S were examined by FESEM. Fig. 5 (a–d) shows the FESEM images of USF, SFT1, SFT2 and NCC–S, respectively. In Fig. 5 (a), it can be seen that the surface of the fiber is smooth due to the presence of wax, oil and lignin [3,53]. Steam explosion in an alkali medium at high temperature decompounds the hemicellulose. It is a high-temperature and pressure process that causes both mechanical and chemical action in the fibers. In alkali treatment assisted with the steam explosion, steam infiltrates the bundles of sisal fibers and causes the removal of impurities like wax and pectin. The steam explosion causes the defibrillation of fibers and enhances hemicellulose hydrolysis [31]. Steam-assisted alkali treatment's main objective is to weaken the intermolecular bonding between the fibrils and increase the rate of hydrolysis of non-cellulosic components in the subsequent reaction. The excess concentration of NaOH in the alkali treatment causes the degradation of cellulose content, leading to a lower yield percentage.

The cracking on the surface of fibers occurs and the suspension of hemicellulose can be seen in Fig. 5 (b). Fig. 5 (c) presents the FESEM image of fibers after bleaching. Most of the lignin was removed by the bleaching and caused further rupturing of fibers. Sodium hypochlorite was used for bleaching to remove lignin. The chlorine present in the sodium hypochlorite solution removed lignin at a very fast rate by oxidation. Hydroxyl, carbonyl, and carboxylic groups are formed during the bleaching, enabling lignin's solubilization in an alkali solution [54]. Acid hydrolysis treatment removes the amorphous region from the bleached fibers because the amorphous region is easily hydrolysed in acid. The concentration of acid and hydrolysis time plays an important role in the shape and size of the extracted nanocellulose [55]. Fahma et al. [56] revealed that the diameter of nanocellulose decreased with the increase in hydrolysis time, but more extensive hydrolysis time decreased the crystallinity of the nanocellulose. The higher concentration of acid causes the burning of the fibers and leaves black residues in the solution [24]. In Fig. 5(d), the FESEM image of NCC-S is shown in which the cylindrical shape and smooth surface are visible.

### 4.5 XRD analysis

**Fig. 6:**
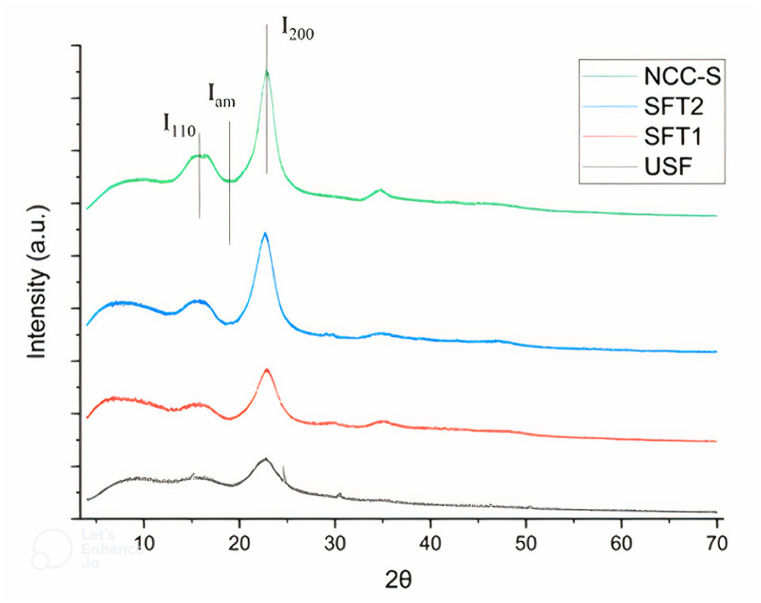
XRD graph of untreated, treatedand nano-crystalline cellulose of sisal fibers from 4° to 70° of 2θ at a wavelength of 1.541 × 10^−10^ m.

Fig. 6 represents the X-ray diffraction analysis of untreated and treated sisal fibers and NCC–S. All the fibers and NCC–S have similar diffraction patterns with two prominent reflection peaks at around 23° and 16° of 2. The highest values of intensities (I_200_) for USF, SFT1, SFT2, and NCC–S were observed at approximately 22.85°, 22.85°, 22.53°, and 22.72° respectively, while the maximum values of I_am_ were observed at around 19.18°, 19.13°, 18.55°, and 18.92° respectively of 2. As a result of the processing, NCC–S showed the highest crystallinity index, followed by treated and untreated fibers. There were also shoulder peaks observed at around 16° of 2 for all the fibers, indicating that cellulose structure had been increased credited to chemical processing and mechanical grinding.

Both amorphous and crystalline regions are present in sisal fibers, but the non–crystalline portion is not entirely disordered, and the crystalline region is not fully crystal. The amorphous part of the cellulose could be removed by employing different treatments, resulting in an improved crystallinity index and degree of crystallinity [57,58]. The crystallinity index was found to be 48.74 % in USF, 64.62 % in SFT1, 72.60 % in SFT2, and 73.54 % in NCC–S. It can be concluded that the crystallinity index is continuously increased from USF to NCC-S due to the removal of non-cellulosic and amorphous components (i.e., hemicelluloses and lignin) after each treatment. Thus, the removal of the non-crystalline region after treatment has increased the crystallinity index from 48.74 % to 73.54 %. Similar behavior can also be seen in the case of the degree of crystallinity. The measured crystallinity degree values for USF, SFT1, SFT2, and NCC–S were 0.66, 0.73, 0.78, and 0.79, respectively. Deepa et al. [45] reported the crystallinity index for various fibers: 80.9 % for banana rachis, 91.3 % for sisal, 86.5 % for kapok, 92.3 % for pineapple leaf, and 84.5 % for coir.

### 4.6 FTIR analysis

**Fig. 7:**
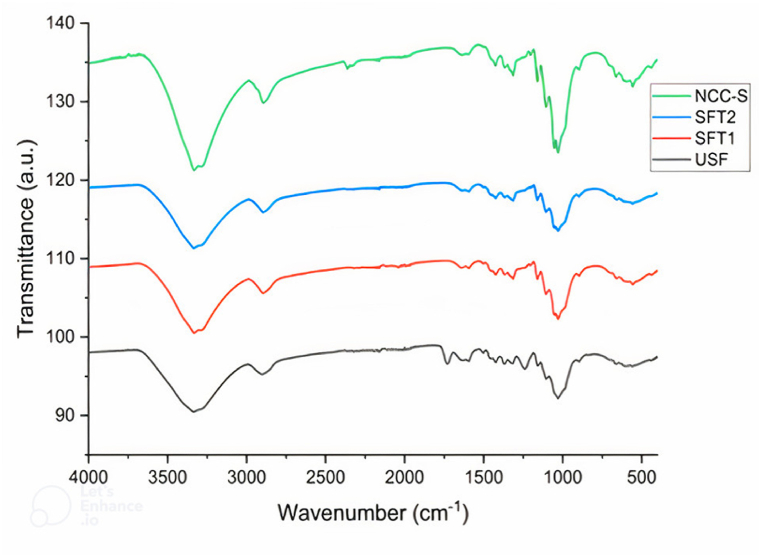
FTIR spectrum of untreated, treated and nano-crystalline cellulose of sisal fibers at 400–4000 cm^−1^ wavelength range.

FTIR analysis was performed to observe the effect of chemical treatments and mechanical grinding on the chemical structure of sisal fibers by eliminating various functional groups. Sisal fibers comprise three materials-cellulose, hemicellulose, and lignin; all these materials have alkanes, esters, ketones, aromatic and alcohols with different oxygen content functional groups. There are majorly two absorption spectra observed: the first one at low wavelength spectra (560–1731 cm^−1^) and the other at high wavelength spectra (2912–3342 cm^−1^), as shown in Fig. 7 [20]. Wide absorption bands in the 4000–2990 cm^−1^ were detected for all the fibers and NCC-S showed the hydroxyl group's OH stretching vibration in all three cellulose, hemicellulose, and lignin. The absorption band among 2880–2990 cm^−1^ was visible in the spectra of all the fibers and NCC-S representing the H–*C*–H group of all three cellulose, hemicellulose and lignin. It was noticed that the peaks at 1732 cm^−1^ because of the C=O stretching vibration of acetyl of lignin and hemicellulose were only present in the spectrum of untreated fibers and removed entirely in the treated fibers and NCC-S.

After the alkali treatment, the peak at 1732 cm^−1^ was almost removed because of the removal of hemicellulose from the fibers. It can also be visualized from FESEM analysis that the surface of fibers turned rough and were defibrillated. The absence of a peak at around 1235–1240 cm^−1^ also indicates the removal of hemicellulose from the fibers. However, this peak was present in the spectrum of untreated fibers because of the *C*–O stretching vibration of hemicellulose in the fibers [23]. The peaks at 1506 cm^−1^ because of the C=O stretching aromatic rings of lignin become lesser after alkali treatment and bleaching and completely removed in the spectrum of NCC-S [59]. The fibers turned white after the bleaching, indicating the removal of lignin from the fibers. The peaks at 1640 cm^−1^ represent the bending vibration of absorbed moisture in the spectrum of untreated fibers, which has been reduced after each treatment suggestingthat water uptakewas reduced in treated fibers and extracted nanocellulose [53]. This characterization reveals that each chemical treatment significantly impacts the chemical structure and morphology of the fibers. The hemicellulose and lignin were completely removed after this process which suggests that the present process is very useful in the extraction of nanocellulose.

#### 4.6.1 Antibacterial analysis

**Fig. 8:**
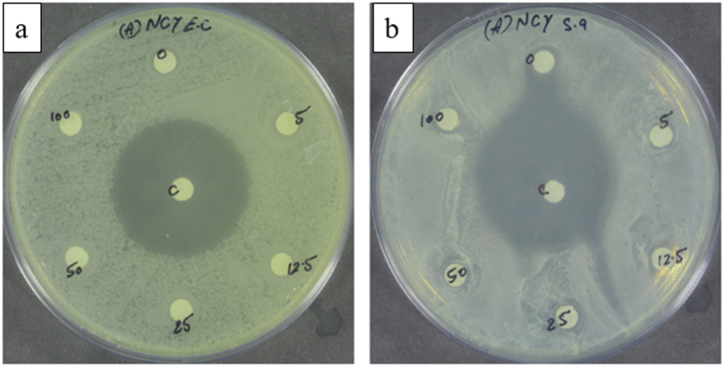
Antibacterial analysis of nano-crystalline celluloseby disc diffusion methodagainst: (a) *E. coli*, and (b) *S. aureus.*

The disc diffusion method was used to measure the antibacterial analysis of nanocellulose against gram–negative bacteria *E. coli* and gram-positive bacteria *S. aureus.* First of all, Mueller Hinton Agar (MHA) plates were spread in inoculated with 100 μl of log cultures of *E. coli* and *S. aureus*. The discs loaded with 10 μl of nanocellulose dispersion prepared in DMSO (Dimethyl sulfoxide) solvent were placed in MHA plate inoculated with culture plate bacteria. One disc was loaded with solvent alone, which served as vehicle control and the ciprofloxacin disc (20 μg) was taken as a positive control. The *E. coli* and *S. aureus* plates were incubated at 37 °C for 24 h and incubation zones around discs were measured. In Fig. 8 (a) and (b), vehicle control is shown with 0, 5, 12.5, 25, 50, and 100, representing NCC-S concentration in mg/ml on ciprofloxacin disc, and C shows the positive control. After 24 h, it was observed that the paper disc impregnated with NCC-S could not control the bacterial growth, as seen by the absence of a halo of growth inhibition surrounding the paper disc. Finally, it can be concluded that the poor antibacterial property of the present NCC-S might be the result of the complete removal of lignin by chemical treatments. Valencia et al. [59] also observed in their study that nanocellulose did not affect the development of any bacterial activity in the absence of residual lignin.

### 4.7 Aerogel analysis

#### 4.7.1 Morphology analysis

**Fig. 9:**
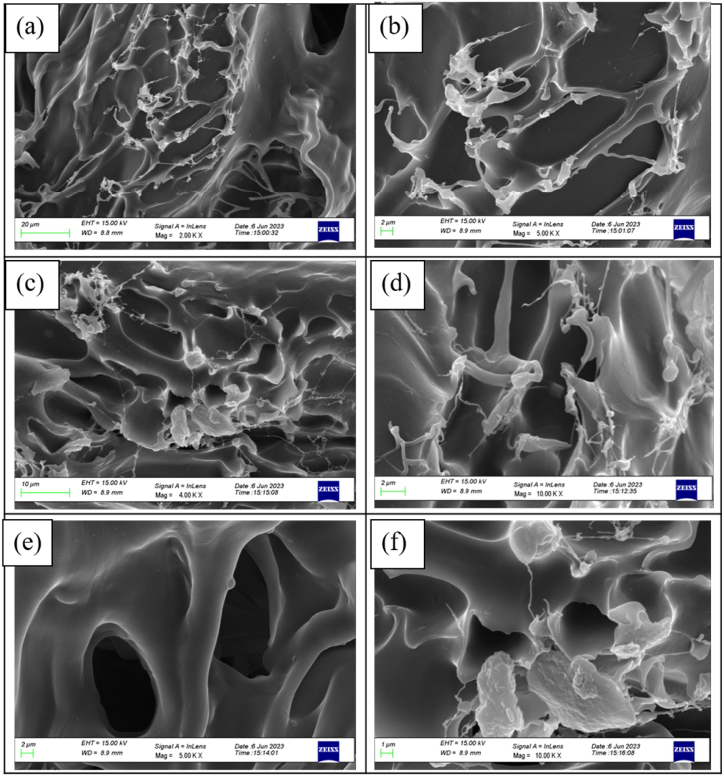
Morphology analysis of aerogel through FESEM at different magnifications.

The porosity and density of aerogel prepared using extracted nanocellulose and chitosan were observed as 95.1 % and 0.075 g/cm^3^ respectively. The values of porosity and density of the present aerogel were found to be comparable with other earlier-developed nanocellulose-based aerogel by many researchers. Table 1 shows the comparison of the porosity and density of the present aerogel with the published results. The surface morphology of aerogel at different magnifications was also investigated through FESEM analysis, as shown in Fig. 9. The morphological analysis reveals that prepared aerogel has a good cross-linking network structure and porosity.

**Table 1:** Comparison of properties of the present aerogel with published results.

Composition/wt. %	Porosity (%)	Density (g/cm^3^)	Processing method	Ref.
CNF/chitosan (40/60)	92.7	0.111	Vacuum Freeze-drying	[39]
CNF/chitosan (60/40)	97.2	0.043	Vacuum Freeze-drying	[39]
PVA/CNF (100/100)	99.5	0.0065	Vacuum Freeze-drying	[44]
CNF/chitosan (100/100)	98.7	0.0186	Vacuum Freeze-drying	[40]
CNF (0.6–1.2 wt %)	99.1–99.5	0.008–0.0138	Chemical vapor deposition	[60]
CNC/chitosan (50/50)	95.1	0.075	Vacuum Freeze-drying	Present work

#### 4.7.2 FTIR

**Fig. 10:**
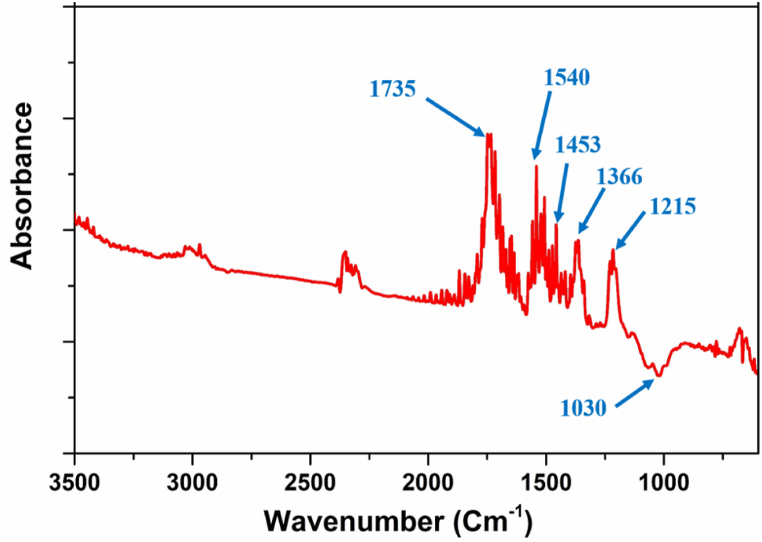
FTIR spectrum of aerogel prepared from NCC-S and chitosan.

The cross-linking structure of aerogel was also confirmed through the FTIR spectra, as shown in Fig. 10. This analysis reveals the various structural features of the aerogel consisted of different functional groups as ketones, alkenes, alcohol, esters and aromatics. The absorption peaks present at around 1735 and 1540 cm^−1^ was due to stretching vibration of carbonyl and amino groups [61]. The peaks present in the absorption band 1735–1215 cm^−1^ might be due to stretching vibration of C=O [62]. The peaks present in the region 1453–1366 cm^−1^ was due to stretching vibration of *C*–O and bending vibration of *N*–H. The absorption peak near 1030 cm^−1^ was observed due to stretching vibration of *C*–*O*–H [63]. In addition to this, sharp peak present at 1453 cm^−1^ also signifies the presence of symmetric CH_2_ bending vibration in the aerogel [62]. Based on the comparison of these results with the results reported in the literature, the good cross-linking network in the aerogel can be ensured [[61], [62], [63]]. Finally, it can be concluded that an aerogel of good cross-linking network, porosity and density was successfully developed using extracted NCC-S with the help of chitosan.

#### 4.7.3 TGA analysis

**Fig. 11:**
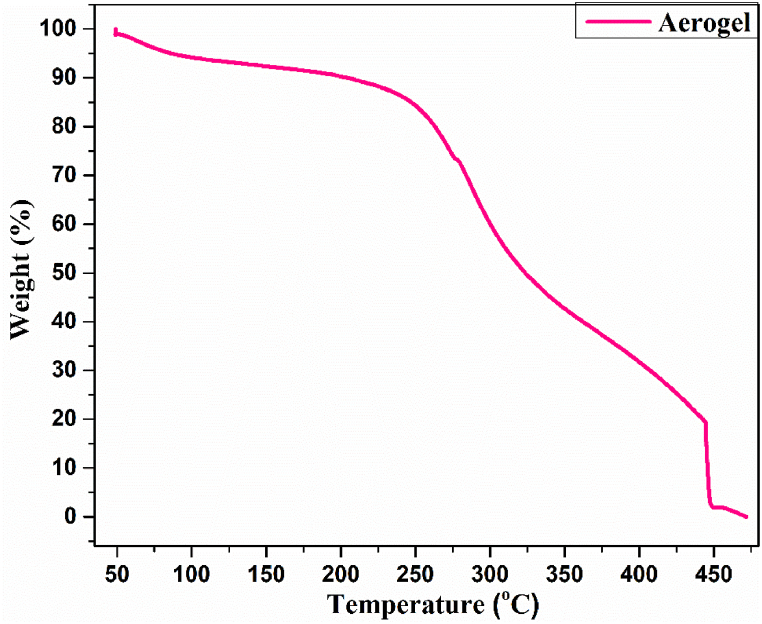
TGA analysis of aerogel.

Thermal behavior of aerogel was investigated by TGA analysis, as shown in Fig. 11. Initially, slightly weight loss of aerogel (less than 10 %) was noticed at 100 °C due to the evaporation of moisture content and volatile compounds present in the aerogel [64]. TGA analysis shows that the aerogel has two onset temperatures first one at 249.3 °C and other at 336.6 °C. The highest thermal degradation temperature of aerogel was observed as 270.5 °C. It was observed that aerogel was majorly thermal decomposed around at 450 °C. The results obtained in the present work are very close and similar to the results reported by Rizal et al. [39] and Zhang et al. [65] in their thermal analysis of the aerogel prepared CNF/chitosan hybrid aerogel. These analysis exhibit that aerogel was successfully prepared from CNC/chitosan and have good thermal stability.

## 5 Conclusion

Nanocellulose in the form of nano-crystalline cellulose has been successfully extracted by the process followed in the present study. The TEM and DLS results confirmed that the extracted nanocellulose particles have a diameter in the nano range. A very high aspect ratio of around 20 was observed for extracted nanocellulose which shows an effectiveness of the present extraction process. This study also suggests that the present method can be utilized as an eco-friendly and efficient method for extracting nano-cellulose from biomass. The FESEM images showed how the surface of the fibers changed after each treatment. FTIR spectra of the fibers and nanocellulose revealed information about the changes in the chemical structures during the process and confirmed the completed elimination of the hemicellulose and lignin for extracted nanocellulose. XRD analysis gave information about the crystallinity index which was found to be increased from 48.74 % in USF to 73.54 % in NCC–S. Further, no antibacterial potential was shown by the present nanocellulose due to the complete removal of lignin. The aerogel prepared from NCC–S/chitos has porosity and density of 95.1 % and 0.075 g/cm^3^. The prepared aerogel is capable to offer good cross-linking network structure and thermal stability. Based on these results, the present aerogel can be utilized as tissue engineering scaffolds, separable membranes and absorbent for oil adsorption from water.

## Additional information

No additional information is available for this paper.

## Data availability statement

Data included in article/supplementary material/referenced in article.

## CRediT authorship contribution statement

**Yash Vishnoi:** Writing – review & editing, Writing – original draft, Visualization, Validation, Methodology, Investigation, Funding acquisition, Formal analysis, Data curation, Conceptualization. **Alok Kumar Trivedi:** Writing – review & editing, Writing – original draft, Visualization, Validation, Methodology, Investigation, Formal analysis, Data curation, Conceptualization. **M.K. Gupta:** Writing – review & editing, Writing – original draft, Visualization, Validation, Supervision, Software, Resources, Project administration, Methodology, Investigation, Funding acquisition, Formal analysis, Data curation, Conceptualization. **Harinder Singh:** Writing – review & editing, Writing – original draft, Visualization, Validation, Resources, Methodology, Investigation, Formal analysis, Data curation, Conceptualization. **Sanjay Mavinkere Rangappa:** Writing – review & editing, Writing – original draft, Visualization, Validation, Supervision, Software, Resources, Methodology, Investigation, Formal analysis, Data curation, Conceptualization. **Suchart Siengchin:** Writing – review & editing, Visualization, Validation, Methodology, Formal analysis, Data curation, Conceptualization.

## Declaration of competing interest

The authors declare that they have no known competing financial interests or personal relationships that could have appeared to influence the work reported in this paper.
